# BCR-ABL1 transcript decline ratio combined BCR-ABL1^IS^ as a precise predictor for imatinib response and outcome in the patients with chronic myeloid leukemia

**DOI:** 10.7150/jca.38752

**Published:** 2020-02-03

**Authors:** Zhimei Cai, Xiting Jia, Jie Zi, Huihui Song, Shujun Wang, Mary McGrath, Lidong Zhao, Chunhua Song, Zheng Ge

**Affiliations:** 1 Department of Hematology, Zhongda Hospital, School of Medicine, Southeast University, Nanjing, Jiangsu, China; 2 Department of Hematology, The First People's Hospital of Lianyungang, The Affiliated Lianyungang Hospital of Xuzhou Medical University, Lianyungang, Jiangsu, China; 3 Hershey Medical Center, Pennsylvania State University College of Medicine, 500 University Drive, Hershey, PA 17033, USA

**Keywords:** chronic myeloid leukemia, imatinib, BCR-ABL1, decline

## Abstract

**Purpose**: The early BCR-ABL1 reduction had the prognostic impact of the chronic-phase chronic myeloid leukemia (CML-CP) patients. This study was to find a more precise early prognosis index at 3 months in the patients with newly diagnosed CML-CP, especially for the patients with BCR-ABL1^IS^ >10%.

**Methods**: We retrospectively analyzed the data of 79 newly diagnosed CML-CP patients from October 2013 to April 2017. All patients took imatinib regularly and continuously and monitored BCR-ABL1 transcript level at baseline and 3, 6, 9, 12, 18 months after starting imatinib treatment.

**Results**: Among the 44(55.7%) patients with BCR/ABL1^IS^ ≤10% at 3 months after imatinib treatment, 12(27.3%) cases did not achieve major molecular response (MMR) at 12 months, and 7(14.9%) patients with the halving time BCR-ABL1 transcript ≤40 days failed to achieve MMR at 12 months. However, approximately twenty-six percent of the patients with BCR-ABL1^IS^ >10% still obtained MMR. Moreover, the patients with BCR-ABL1^IS^ ≤10% and halving time ≤40 days had a significantly better MMR than that of the patients with the BCR-ABL1^IS^ ≤10% and halving time >40 days (88.6% versus 11.1%, *P* <0.001). However, the patients with the BCR-ABL1^IS^ >10% and halving time >40 days rarely achieved MMR at 12 months.

**Conclusion**: These data indicated that the halving time of BCR-ABL1 transcript was also an important prognostic factor as that of the BCR-ABL1^IS^. Combined observations of these two prognosis indexes are more accurate predictor for the long-term molecular response, especially for the CML-CP patients with BCR-ABL1^IS^ >10%, and which is helpful for TKI switching as early as possible to improve patients' survival and reduce drug costs.

## Introduction

The outcome of patients with chronic-phase chronic myeloid leukemia (CML-CP) has been radically changed with the use of the first-generation tyrosine kinase inhibitor (TKI)-imatinib, but a small number of patients still progress. The National Comprehensive Cancer Network (NCCN) and European Leukemia Net (ELN) recommendations have incorporated the early molecular response (EMR) into the definitions of optimal response^1^, ^2^. Evaluation of EMR can guide early therapy switch, thus improve the long-term survival of CML patients. International experts have also identified the importance of EMR^3^-^6^. BCR-ABL1^IS^ ≤10% at 3 months was defined as the EMR^7^, ^8^. Baccarani M et al^9^ and Jain P et al^10^ presented that the measuring BCR-ABL1^IS^ at 3 months was the only factor to predict the outcome. Furthermore, the NCCN guidelines recommended that the patients with EMR failure at 3 months of imatinib therapy switched to the other TKIs for the maximum benefit^2^. For this reason, at present EMR is a main early prediction index, but some clinical data are not consistent with it. Hence it is imperative to find a more accurate early prediction index.

The ELN suggested deciding if the patient needs to switch until the 6-month of the imatinib failure 1. Moreover, not all patients who did not reach EMR had poor prognosis, and they could be distinguished by the ratio decline from baseline, rather than by one single measurement of BCR-ABL1^IS^ at 3 months 11. Additionally, the number of BCR-ABL1^IS^ was diverse in each newly diagnosed CML patient with the inconsistent treatment compliance and the different toxic and side effects, which resulted in a large gap of gene copy numbers at 3 months of treatment. The prognosis may not be precise based on BCR-ABL1^IS^ alone. A few studies have focused on the rate of early BCR-ABL1 transcript elimination 12-14. However, research in this area was still a pioneer, and their reports were inconsistent with each other. Moreover, there are no reports concerning the combination of BCR-ABL1^IS^ and the BCR-ABL1 halving time at 3 months for EMR evaluation, especially for the CML-CP patients with BCR-ABL1^IS^ >10%.

The purpose of this study was to retrospectively analyze the clinical feature, gene level and survival status of 79 patients with newly diagnosed CML-CP and assessed the relationship between the halving time threshold and BCR-ABL1^IS^ at 3 months with molecule response, overall survival (OS), progression-free survival (PFS) and event-free survival (EFS). This study firstly proposed a better, more precise early prognosis index at 3 months, especially for the CML-CP patients with BCR-ABL1^IS^ >10%, which consequently guide the treatment switching in time to improve the level of molecular response and quality of life of patients.

## Patients and Methods

### Patients

The study retrospectively analyzed the data of newly diagnosed CML-CP patients from the department of hematology, Affiliated Zhongda Hospital of Southeast University and The First People's Hospital of Lianyungang, China. Inclusion criteria were: firstly, the newly diagnosed CML-CP patients without previous treatment; secondly, the patients treated with imatinib (Chia Tai Tianqing Pharmaceutical Group Co., Ltd., Lianyungang, Jiangsu province, China); thirdly, the patients were followed for a minimum of 18 months; fourthly, the patients underwent molecular monitoring every three months during the first year of treatment or achieving major molecular response (MMR). Exclusion criteria were: firstly, the patients who did not take imatinib regularly and continuously; secondly, the patients who delayed in molecular monitoring or with the BCR-ABL1 kinase domain mutation; thirdly, the patients with severe cardio-cerebrovascular disease and/or mental illness; fourthly, the pregnant patients. The detailed clinic parameters of enrolled pateints are also described in Table 1.

### Assessment of treatment response and molecular analysis

All patients took imatinib regularly and continuously, and the dose could be adjusted according to the patient's reaction. Bone marrow samples of the patients were obtained at baseline and 3, 6, 9, 12, 18 months after starting imatinib treatment, and the BCR-ABL1 transcript level was detected by quantitative real-time polymerase chain reaction (QR-PCR) for molecular efficacy evaluation, and ABL1 as the control gene, which had been studied extensively for suitability for BCR-ABL1 measurement^4^, ^8^. The final results were expressed as BCR-ABL1/ABL1 ratios in percentages according to the international scale (IS) (BCR-ABL1^IS^ = BCR-ABL1 transcript number/ABL1 transcript number × 100% × conversion coefficient, conversion coefficient: 0.96)^7^, ^10^, ^12^. Currently, the treatment of CML-CP emphasized molecular response monitoring, which included MMR and deep molecular response (DMR). MMR was defined as a 3-log reduction of BCR-ABL1 transcript levels, corresponding to ≤0.1% BCR-ABL1^IS^. DMR was defined as a 4-log reduction of BCR-ABL1 transcript levels, corresponding to ≤0.01% BCR-ABL1^IS^ 14. Survival assessment for CML treatment included OS, PFS and EFS. OS was defined by absence of death for any reason, PFS by absence of accelerated phase (AP), blast phase (BP) and death for any reason; EFS was measured from the start of treatment to the date of any of the following events: treatment failure using the 2013 ELN recommendation definitions, progression to AP or BP, death from any cause, or reasons for changing treatment other than toxicity 10.

### Calculation of the halving time of BCR-ABL1 transcript with imatinib treatment

The halving time for BCR-ABL1 transcript was calculated as A × 3log_10_2/log_10_ (B/C), where A = the actual treatment time in days, from the day of the initial evaluation of BCR-ABL1 before treatment to the evaluation for BCR-ABL1 transcript at 3 months; B = BCR-ABL1 transcript value at diagnosis; and C = BCR-ABL1 transcript value at 3 months 15, 16.

### Statistical analysis

Fisher's exact test was constructed to determine associations between categorical variables. OS, PFS, and EFS were estimated using the Kaplan-Meier method. The log-rank test was used to identify the significant differences between curves. The statistical significance was defined as *P* < 0.05. All calculations were performed with the SPSS software Version 24.0.

## Results

The study analyzed the data of 116 newly diagnosed CML-CP patients collected from October 2013 to April 2017. Among them, 37 patients were excluded in the data analysis because of delaying in molecular monitoring or with the BCR-ABL1 kinase domain mutation. Therefore, there were 79 patients included in the analysis. The patient characteristics were described in Table 1. The median age of the patients (48 male and 31 female) at diagnosis was 44 years (range, 13-76 years) (Table 1). The molecular response of the female group was slightly higher than that of the male, but the statistical significance was not satisfied (MMR: 12months: 58.1% versus 47.9%, *P* =0.483, 18months: 61.3% versus 50.0%, *P* =0.033; DMR: 12months: 22.6% versus 6.3%, *P* =0.325; 18months: 32.3% versus 16.7%, *P* =0.107). The 39, 23, and 17 patients were classified as low, intermediate, and high-risk group, respectively, based on the Sokal prognostic scoring system. The molecular response of the low-risk group was higher than that of the intermediate/high-risk group at 12 and 18 months after treatment, but not all the data of each group had statistically significant (MMR: 12months: 61.5% versus 42.5%, *P* =0.090, 18months: 66.7% versus 42.5%, *P* =0.031; DMR:12months: 12.5% versus 12.8%, *P* =0.966; 18months: 28.2% versus 17.5%, *P* =0.257). Then, the patients were grouped into 3 risk groups according to Hasford prognostic scoring system, which resulted in 49 low-risk patients, 26 medium-risk patients, and 4 high-risk patients. A significant difference in the MMR was observed among the low-risk group and the intermediate/high-risk group (12months: 61.2% versus 36.7%, *P* =0.034; 18months: 65.3% versus 36.7%, *P* =0.013) but no statistical significance in DMR was observed in the two groups (12months: 14.3% versus 10.0%, *P* =0.734; 18months: 24.5% versus 20.0%, *P* =0.644).

**Figure 1:**
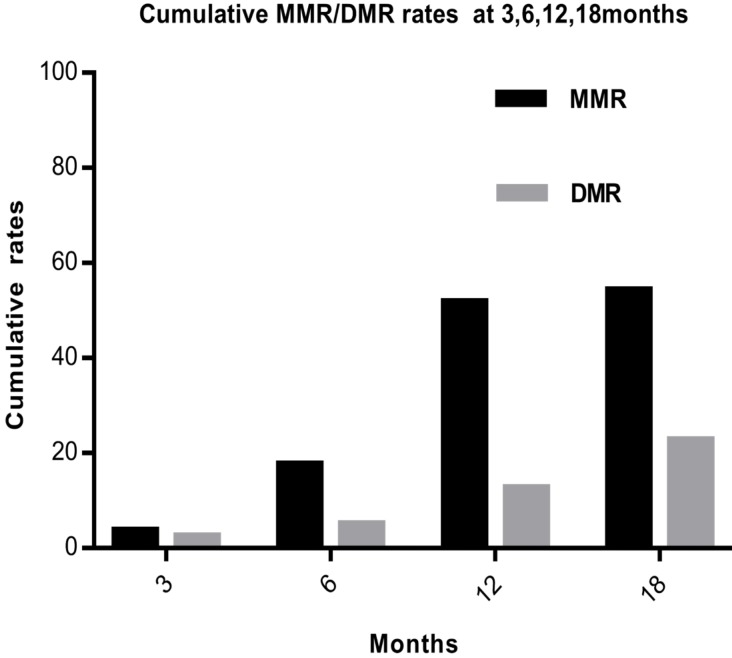
** The overall cumulative MMR and DMR rates at 3, 6, 12 and 18 months.** Abbreviations: MMR: major molecular response, DMR: deep molecular response

Based on the molecular evaluation, 44 of the 79 (55.7%) patients achieved 10% of BCR-ABL1^IS^ by 3 months, 31 of the 79 (39.2%) achieved 1% by 6 months, and 41 of the 79 (51.9%) achieved 0.1% by 12 months. The overall cumulative MMR rates were 3(3.8%), 14(17.7%), 41(51.9%), and 43(54.4%) at 3, 6, 12, and 18months, respectively. The 3-, 6-, 12-, and 18-month DMR rates were 2(2.5%), 4(5.1%), 10(12.7%) and 18(22.8%), respectively (Fig. 1).

**Figure 2:**
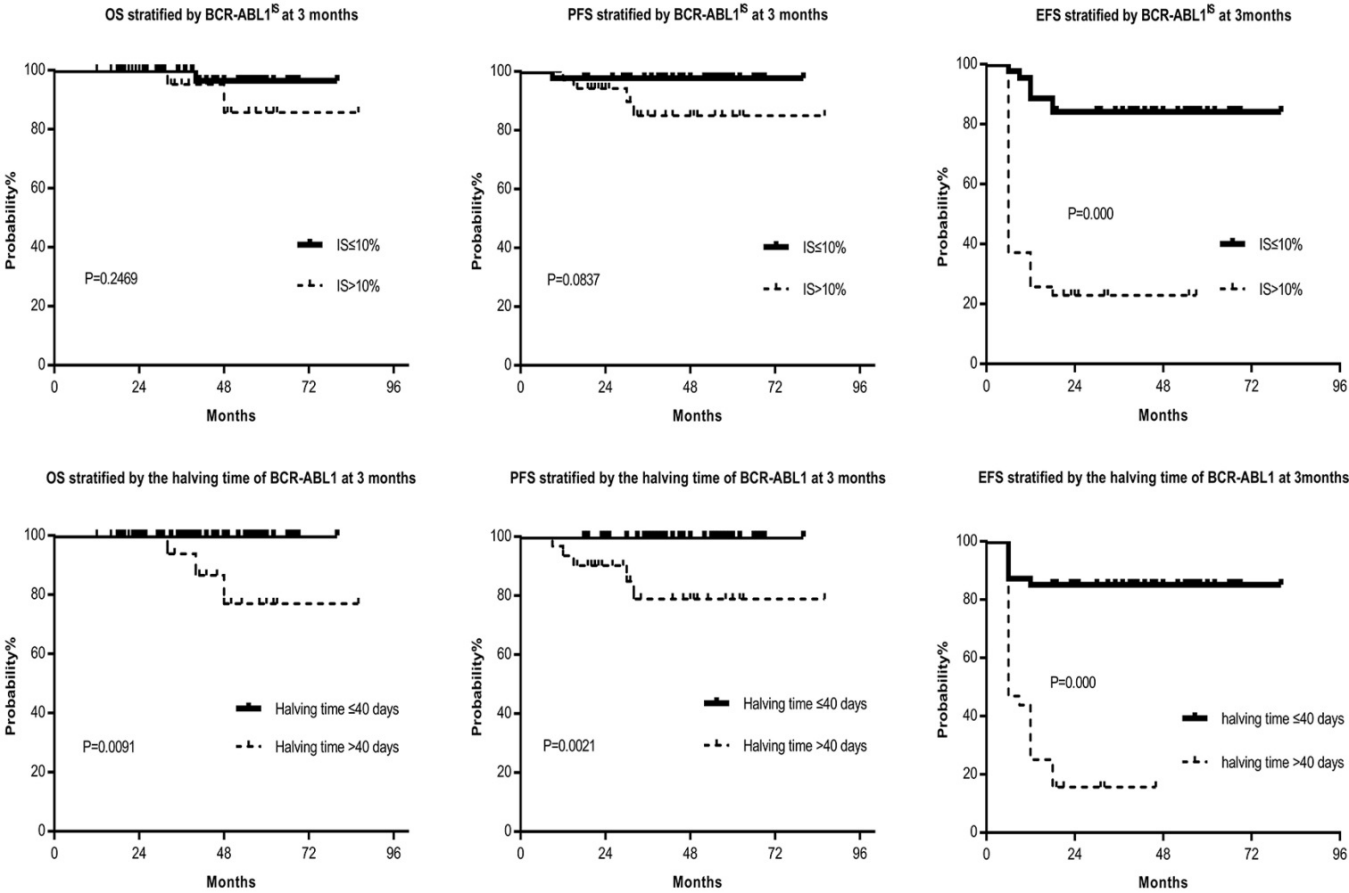
** OS, PFS and EFS stratified by the BCR-ABL1^IS^ and halving time at 3 months, respectively.** Figure 2 showed that the OS, PFS, and EFS of the BCR-ABL1^IS^ ≤10% group were superior to that of the >10% group, although only the EFS reached statistical significance. Furthermore, the OS, PFS, and EFS of the halving time ≤40 days group were significantly higher than that of the >40 days group, and there were statistically significant differences between each group. Abbreviations: OS: overall survival, PFS: progression-free survival, EFS: event-free survival

Among the 44 patients with BCR/ABL1^IS^ ≤10% at 3 months after imatinib treatment, 32(72.7%) cases achieved MMR at 12 months, and 12(27.3%) cases failed in molecular remission at 12 months. Approximately twenty-six percent of the patients with BCR-ABL1^IS^ >10% still obtained MMR. Comparison of MMR and DMR in the patients with BCR-ABL1^IS^ ≤10% or that >10% were as follows: 1-year MMR, 72.7% versus 25.7%, *P* =0.000; 1-year DMR, 15.9% versus 8.6%, *P* =0.499; 1.5-year MMR ratio, 75.0% versus 28.6%, *P* =0.000; 1.5-year DMR ratio, 34.1% versus 8.6%, *P* =0.008; 5 years OS, 97.7% versus 94.3%, *P* =0.2469; 5 years PFS, 97.7% versus 88.6%, *P*=0.0837; 5 years EFS, 84.1% versus 22.9%, *P* =0.000 (Table 2, Fig. 2). Compared to the BCR-ABL1^IS^ >10% group, the molecular response of BCR-ABL1^IS^ ≤10% group was significantly increased, also the OS, PFS, and EFS were increased, although only the EFS reached statistical significance. Furthermore, the patients with BCR-ABL1^IS^ >10% at 3 months did not reach the optimal response, but not all patients fail to the therapy, and 9/35(25.7%) patients subsequently achieved MMR at 12 months. One characteristic shared by those patients was all of them had the halving time of BCR-ABL1 transcript ≤40 days. Therefore we investigated the association between the halving time of BCR-ABL1 transcript and obtaining MMR by 12 months and determined the optimal halving time threshold was 40 days.

Furthermore, the patients were classified into two groups, the halving time of BCR-ABL1 transcript ≤40 days and that >40 days, and 47(59.5%) patients with the halving time BCR-ABL1 transcript ≤40 days and 32(40.5%) patients with that >40 days at 3 months. Regarding molecular assessment, 40 of 47 (85.1%) patients with the halving time BCR-ABL1 transcript ≤40 days achieved MMR at 12 months. However, only 1 of 32 (3.1%) patients with the halving time BCR-ABL1 transcript >40 days obtained MMR. Comparing MMR and DMR in the patients with halving time BCR-ABL1 transcript ≤40 days versus that >40 days, it showed: 1-year MMR, 85.1% versus 3.1%, *P* =0.000; 1-year DMR, 21.3% versus 0%, *P* =0.005; 1.5-year MMR, 85.1% versus 9.4%, *P* =0.000; 1.5-year DMR, 36.2% versus 3.1%, *P* =0.001; 5 years OS, 100% versus 90.6%, *P* =0.0091; 5 years PFS, 100% versus 84.4%, *P* =0.0021; 5 years EFS, 85.1% versus 15.6%, *P* =0.000 (Table 2, Fig. 2). The molecular response, OS, PFS, and EFS of the halving time ≤40 days group were significantly higher than that of the >40 days group.

**Figure 3:**
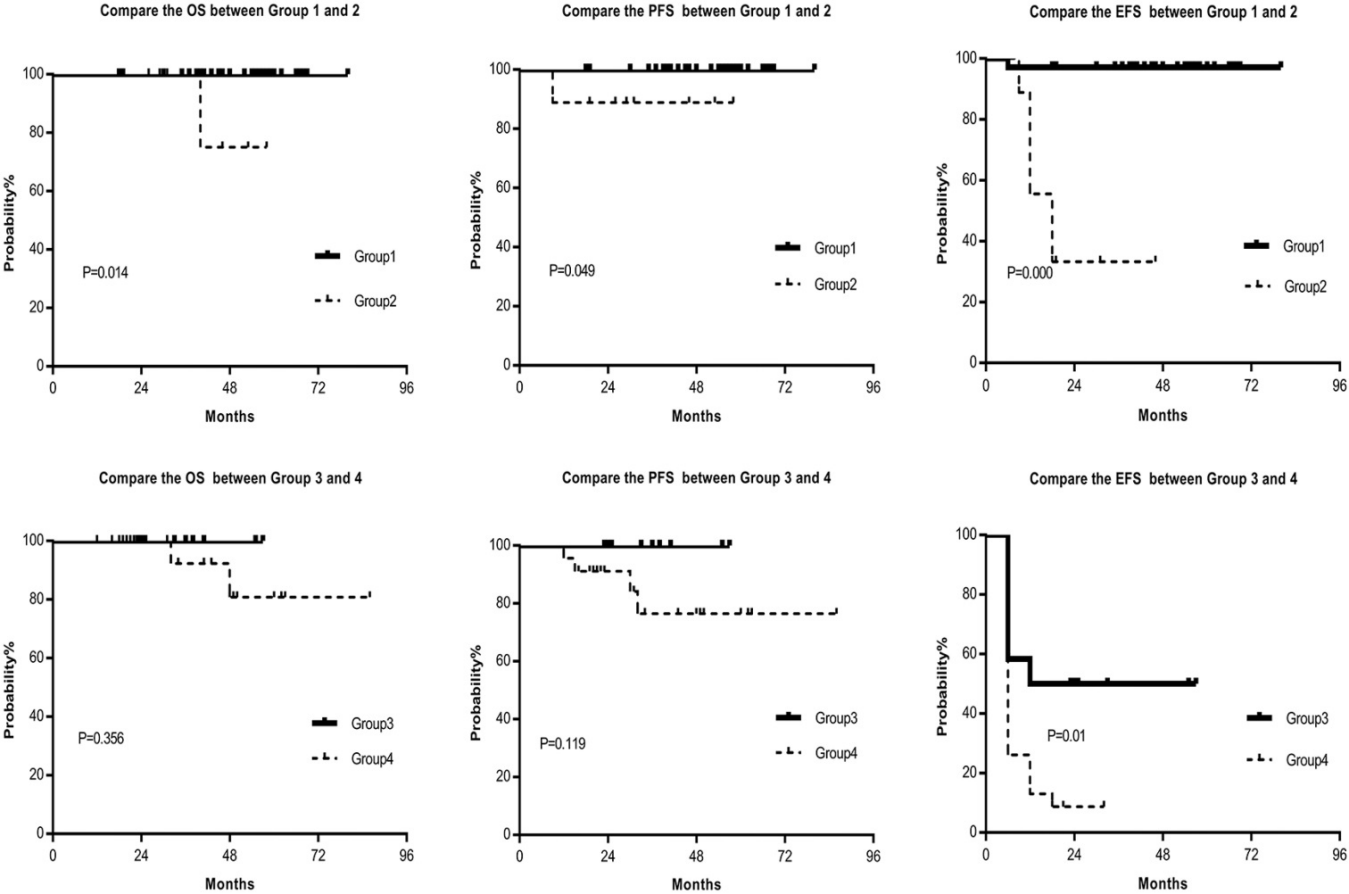
** Compare the OS, PFS and EFS between Group 1 and Group 2, Group 3 and Group 4, respectively.** Group1 had considerably better OS, PFS and EFS than Group 2, and the differences were statistically significant. Moreover, the OS, PFS, and EFS of Group 4 were notably worse than that of Group 3; and a statistically significant difference in EFS was observed. Abbreviations: OS: overall survival, PFS: progression-free survival, EFS: event-free survival, Group 1: the patients with BCR-ABL1^IS^ ≤10% and halving time of BCR-ABL1 transcript ≤40days, Group 2: the patients with BCR-ABL1^IS^ ≤10% and halving time >40 days, Group 3: the patients with BCR-ABL1^IS^ >10% and halving time ≤40 days, Group 4: the patients with BCR-ABL1^IS^ >10% and halving time >40 days.

Based on our analysis, the CML patients were then classified into the following four groups. Group 1: the patients with BCR-ABL1^IS^ ≤10% and halving time of BCR-ABL1 transcript ≤40days; Group 2: the patients with BCR-ABL1^IS^ ≤10% and halving time >40 days; Group 3: the patients with BCR-ABL1^IS^ >10% and halving time ≤40 days; Group 4: the patients with BCR-ABL1^IS^ >10% and halving time >40 days. We analyzed the survival of the patients in the four groups and found that Group1 had considerably best MMR, DMR, OS, PFS and EFS. Moreover, the differences were statistically significant between the first group and the second group (Table 3, Fig. 3). Furthermore, the molecular response, OS, PFS, and EFS of the fourth group were notably worse than that of the third group; and statistically significant differences in MR and EFS in the two groups were observed (Table 3, Fig. 3). Our data suggested that the halving time of BCR-ABL1 transcript was an important prognostic indicator as the BCR-ABL1^IS^, and the combination of BCR-ABL1^IS^ and halving time of BCR-ABL1 transcript is a better prognostic indicator.

Drug-related adverse effects were tolerable. The most common toxicity was non-hematological toxicity, with an incidence of about 59%, which was similar to previous reports 14. Non-hematologic toxicities mainly included fluid retention, rash, muscle cramps, gastrointestinal upset, and jaundice. Hematologic toxicities were concentrated in level 1-3^17^. These reactions gradually disappeared with the prolongation of treatment time, and no patient discontinued due to the toxic reaction.

## Discussion

This study confirmed the early prognostic value of both halving time of BCR-ABL1 transcript and BCR-ABL1^IS^ at 3 months of imatinib treatment. This research also presented that it was not precise to predict molecules response using BCR-ABL1^IS^ or halving time alone. Our data suggested combining two indicators is a more efficient and precise way for prognostic prediction.

The NCCN and ELN demonstrated the importance of early molecular reactions 3, 5, 18. Some reports indicated that CML-CP patients who achieved BCR/ABL1^IS^ ≤10% at 3 months had a favorable outcome^19^; and some reports showed the CML-CP patients with low BCR-ABL1 transcript levels at diagnosing were easier to achieve molecular response^1^, ^20^. But recent studies observed no significant association between BCR-ABL1 transcript levels at first diagnoses and prognosis 1, 21.

Our retrospective analysis found that the early molecular response is valuable for estimating prognosis of CML-CP patients; however the early molecular response did not just refer to the BCR/ABL1^IS^ ≤10% at 3 months, it also referred to the halving time. Our results are consistent with the previous report, which showed the strong predictive value of early molecular reactions included the halving time and 3-month BCR/ABL1^IS^ 18.

The significance of the halving time for prediction of better molecular response and survival were previously reported. Huet et al. 20 found that the patients with the halving time of ≤19 days had a superior molecular response at 12 months than did patients with the halving time of >19 days. Branford 12 demonstrated that the rate of BCR-ABL1 decline from baseline might be a critical prognostic discriminator of the very poor prognosis patients among those who were > 10% at 3 months, and they found that patients with BCR-ABL1 halving time <76 days had significantly superior outcomes compared with patients whose BCR-ABL1 values did not halve by 76 days. Iriyama et al. 14 concluded that patients with BCR-ABL1 halving time ≤14 days were easier to achieve molecular responses by 12 months.

Our study also highlighted that the importance of halving time of early BCR-ABL1 transcript in the patients with CML-CP on outcome prediction. The threshold of BCR-ABL1 halving time in our analysis was also different from the reports, which may be due to the differences in the number of specimens and the study in different regions. Further study is needed to large sample number for standardizing the uniform time of BCR-ABL1 copies decline.

In our study, 27.3% patients with the BCR/ABL1^IS^ ≤10% at 3 months after imatinib treatment did not obtain MMR at 12 months; and about 15% patients with the BCR-ABL1 halving time ≤40 days failed to achieve MMR at 12 months. These data suggested that a single prognostic indicator was inaccurate. More importantly, when the patients were divided into four groups according to the BCR-ABL1^IS^ and halving time, and the results were significantly changed. Approximately 89% of the patients with BCR-ABL1^IS^ ≤10% and halving time ≤40 days at 3 months achieved MMR at 12 months, and these patients achieved the statistically significant higher rate of optimal responses according to the patients with BCR-ABL1^IS^ ≤10% and halving time >40 days. Furthermore, very few patients with the BCR-ABL1^IS^ >10% at 3 months and halving time >40 days (Group 4) achieved an optimal response by 1.5 years (MMR ratio at 1 year: 0%; MMR ratio at 1.5 years: 8.7%). This data suggested the higher value of combination of the two indicators for the patients with BCR-ABL1^IS^ >10%.

Unfortunately, we did not find the data concering the dynamic changes of BCR-ABL1 transcript through database for CML patients in TCGA, cBioPortal, TARGET, GEO, SEER, NCDB, MalaCards, GeneCards, Clinicaltrials, BioLINCC and oncomine databases. However, the National Cancer Institute (NCI)'s Physician Data Query (PDQ) summarized the cancer information about CML-CP treatment and reported that, in a retrospective analysis, even the patients with BCR/ABL1 transcript level greater than 10% after 3 months of therapy did well when the halving time was less than 76 days (all patients enrolled in Australia, New Zealand, Singapore, South Africa, and South America)^12^, and mandating a change of therapy based on this 10% transcript level at 3 to 6 months was problematic because 75% of patients did well even with a suboptimal response^1^. This report is consistent with our data which highlighted the importance of the halving time of BCR-ABL1 transcript in patients with CML-CP on outcome prediction. Our result also suggested that a single prognostic indicator (BCR-ABL1^IS^ or halving time of BCR-ABL1 transcript) was inaccurate, and the combination of the two prognostic indicators was more accurate than angle observation, especially for the CML-CP patients with BCR-ABL1^IS^ >10%.

Owing to imatinib treatment might be different in different race, region and different patient clinical profiles; we will validate our results in much larger multi-center cohorts in the future.

## Conclusion

This study suggests that the BCR-ABL1 halving time and BCR-ABL1^IS^ at 3 months are two early prognostic discriminators for CML-CP patients treated with imatinib. The patients who have the BCR-ABL1 halving time >40 days and BCR-ABL1^IS^ >10% at 3 months will have the poorest outcome, we recommend these patients switching to second-generation TKIs at early time points. Our result firstly suggests that the combination of the two prognostic indicators is more accurate than angle observation, especially for the CML-CP patients with BCR-ABL1^IS^ >10%, and which is helpful for TKI switching as early as possible to reach DMR, improve patients' survival and reduce drug costs.

## Figures and Tables

**Table 1:** Characteristics of CML-CP patients in this study

Factors	N=79
**Age, N**	44 (range 13-76)
**Sex, male, N (%)**	48 (60.8%)
**Sokal risk, N (%)**	
Low-risk	39(49.4%)
Intermediate-risk	23(29.1%)
High-risk	17(21.5%)
**Hasford risk, N (%)**	
Low-risk	49(62.0%)
Intermediate-risk	26(32.9%)
High-risk	4(5.1%)
**Interval since diagnosis, weeks, median (range)**	2(1-40)
**Daily doses of imatinib (mg/day), median (range)**	400(300-600)
**Baseline BCR-ABL1^IS^ (%), median (range)**	65.1 (1.8-667.2)
**Prior treatment**	
None (%)	49(62.0%)

**Table 2:** Probabilities of MMR and DMR at 12, 18 months by univariate analysis of the BCR/ABL1^IS^ and halving time at 3 months

V	NO.	MMR at 12 months	P	DMR at 12 months	P	MMR at 18 months	P	DMR at 18 months	P
IS≤10%	44	32(72.7%)	0.000	7(15.9%)	0.499	33(75.0%)	0.000	15(34.1%)	0.008
IS>10%	35	9(25.7%)		3(8.6%)		10(28.6%)		3(8.6%)	
HT≤40d	47	40(85.1%)	0.000	10(21.3%)	0.005	40(85.1%)	0.000	17(36.2%)	0.001
HT>40d	32	1(3.1%)		0(0.0%)		3(9.4%)		1(3.1%)	

Abbreviations: V: variable, No.: number, MMR: major molecular response, DMR: deep molecular response, P: p value, HT: the halving time, d: days

**Table 3:** Probabilities of MMR and DMR at 12, 18 months by combining observations of the BCR/ABL1^IS^ and halving time at 3 months

G	V	No.	MMR at 12 months	P	DMR at 12 months	P	MMR at 18 months	P	DMR at 18 months	P
1	IS ≤10% and HT ≤40 d	35	31(88.6%)	<0.001*	7(20%)	0.314*	32(91.4%)	<0.001*	15(42.9%)	0.018*
2	IS ≤10% and HT >40 d	9	1(11.1%)		0(0%)		1(11.1%)		0(0%)	
3	IS >10% and HT ≤40 d	12	9(75%)	<0.001^#^	3(25%)	0.034^#^	8(66.7%)	0.001^#^	2(16.7%)	0.266^#^
4	IS >10% and HT >40 d	23	0(0%)		0(0%)		2(8.7%)		1(4.3%)	

Notes: *P value calculated for Group 1 versus Group 2, #P value calculated for Group 3 versus Group 4. Abbreviations: G: group, V: variable, No.: number, MMR: major molecular response, DMR: deep molecular response, P: p value, HT: the halving time, d: days
